# Constitutive or Induced HIF-2 Addiction is Involved in Resistance to Anti-EGFR Treatment and Radiation Therapy in HNSCC

**DOI:** 10.3390/cancers11101607

**Published:** 2019-10-21

**Authors:** Pierre Coliat, Ludivine Ramolu, Jérémie Jégu, Christian Gaiddon, Alain C. Jung, Erwan Pencreach

**Affiliations:** 1 Centre de Lutte Contre le Cancer Paul Strauss, 67200 Strasbourg, France; pcoliat@strasbourg.unicancer.fr (P.C.); lramolu@unistra.fr (L.R.); 2 Service de Pharmacie, Centre de Lutte Contre le Cancer Paul Strauss, 67200 Strasbourg, France; 3 Université de Strasbourg, Inserm, UMR_S1113, 67200 Strasbourg, France; jeremie.jegu@unistra.fr (J.J.); gaiddon@unistra.fr (C.G.); 4 Laboratoire d’Épidémiologie et de Santé Publique, Université de Strasbourg, 67200 Strasbourg, France; 5 Service de Santé Publique, Hôpitaux Universitaires de Strasbourg, 67200 Strasbourg, France; 6 Laboratoire de Biochimie et Biologie Moléculaire, Hôpitaux Universitaires de Strasbourg, 67200 Strasbourg, France

**Keywords:** head and neck squamous cell carcinoma, anti-EGFR targeted therapy, resistance, oncogenic addiction, HIF-2α

## Abstract

Background: management of head and neck squamous cell carcinomas (HNSCC) include anti-Epidermal Growth Factor Receptor (EGFR) antibodies and radiotherapy, but resistance emerges in most patients. *RAS* mutations lead to primary resistance to EGFR blockade in metastatic colorectal cancer but are infrequent in HNSCC, suggesting that other mechanisms are implicated. Since hypoxia and Hypoxia Inducible Factor-1 (HIF-1) have been associated with treatment failure and tumor progression, we hypothesized that EGFR/mammalian Target of Rapamycin (mTOR)/HIF-1 axis inhibition could radiosensitize HNSCC. Methods: We treated the radiosensitive Cal27 used as control, and radioresistant SQ20B and UD-SCC1 cells, in vivo and in vitro, with rapamycin and cetuximab before irradiation and evaluated tumor progression and clonogenic survival. Results: Rapamycin and cetuximab inhibited the mTOR/HIF-1α axis, and sensitized the SQ20B cell line to EGFR-inhibition. However, concomitant delivery of radiation to SQ20B xenografts increased tumor relapse frequency, despite effective HIF-1 inhibition. Treatment failure was associated with the induction of HIF-2α expression by cetuximab and radiotherapy. Strikingly, SQ20B and UD-SCC1 cells clonogenic survival dropped <30% after HIF-2α silencing, suggesting a HIF-2-dependent mechanism of oncogenic addiction. Conclusions: altogether, our data suggest that resistance to EGFR inhibition combined with radiotherapy in HNSCC may depend on tumor HIF-2 expression and underline the urgent need to develop novel HIF-2 targeted treatments.

## 1. Introduction

The overexpression of Epidermal Growth Factor Receptor (EGFR) in >90% of head and neck squamous cell carcinoma (HNSCC) lesions correlates with adverse prognosis [1,2,3,4], and was used as a rationale to evaluate the effect of EGFR-targeted therapies [5]. However, more than 10 years after Food and Drug Administration (FDA)-approval of the use of Cetuximab, an anti-EGFR monoclonal antibody, alone or in combination with either radiotherapy or chemotherapy, HNSCC patient outcome was only moderately improved [6]. The molecular mechanisms that drive head and neck tumor resistance to EGFR-targeted therapies are poorly understood (for review see [7]). Mutations that trigger a constitutive activity of the EGFR receptor (mutations of the EGFR tyrosine kinase domain; expression of the EGFR variant III), or mutations that induce resistance to anti-EGFR antibodies (e.g., RAS genes mutations) are infrequent in HNSCC [8]. In contrast, hypoxia, a common feature of the microenvironment in HNSCC [9], and expression of Hypoxia-Inducible Factor-1 (HIF-1), have been associated with resistance to chemo- and radiotherapy and poorer outcome [10,11]. Hypoxia-related radioresistance has been acknowledged for decades but the specific role of HIF-1, independently of oxygen tension, has also been demonstrated [12], suggesting that HIF-1 inhibition is a promising strategy to radiosensitize tumor cells. While hypoxia is the primary stimulus for HIF-1 upregulation, the constitutive activation of the EGFR/mTOR axis in a majority of lesions contributes to the increased HIF-1α subunit expression through translational regulation [13], and represents a candidate druggable target in HNSCC.

In the present study, we evaluated the interest of inhibiting the EGFR/mTOR/HIF-1 axis to radiosensitize head and neck cancer cells based on combining the anti-EGFR monoclonal antibody cetuximab and the mTOR inhibitor rapamycin. We explored cell response to this combination in vitro and in vivo and identified HIF-2 as a critical factor responsible for tumor resistance and accelerated relapse.

## 2. Results

### 2.1. Epidermal Growth Factor Receptor (EGFR)/mTOR/ Hypoxia Inducible Factor-1 (HIF-1) Axis Inhibition Effectively Reduces Cell Proliferation and Head and Neck Squamous Cell Carcinoma (HNSCC) Tumor Burden

The anti-proliferative activity of the anti-EGFR monoclonal antibody cetuximab and the mTOR inhibitor rapamycin were determined in vitro, in normoxic (20% O_2_) and hypoxic (1% O_2_) conditions, on the cetuximab-sensitive Cal27 and cetuximab-resistant SQ20B cell line models. In each case, 5 nM rapamycin or 2.5 µg/mL cetuximab were the minimal drug concentrations to achieve maximal cell proliferation inhibition (Figure S1A,B). A 5 nM rapamycin and cetuximab co-treatment further impacted the growth of Cal27 and SQ20B cell cultures, with a 50% and 25% decrease of proliferation, respectively (Figure S1C,D). This effect was found to be additive (Table S1). We used phosphorylation of S6RP, which is a downstream effect of mTOR activation, as a molecular read-out for rapamycin and rapamycin/cetuximab treatment efficacy. Interestingly, the anti-proliferative effect of these treatments correlated with decreased phosphorylation of S6RP and decreased HIF-1α protein expression (Figure S1E). Therefore, 5 nM rapamycin and 2.5 μg/mL cetuximab were further used in our in vitro experiments.

**Figure 1:**
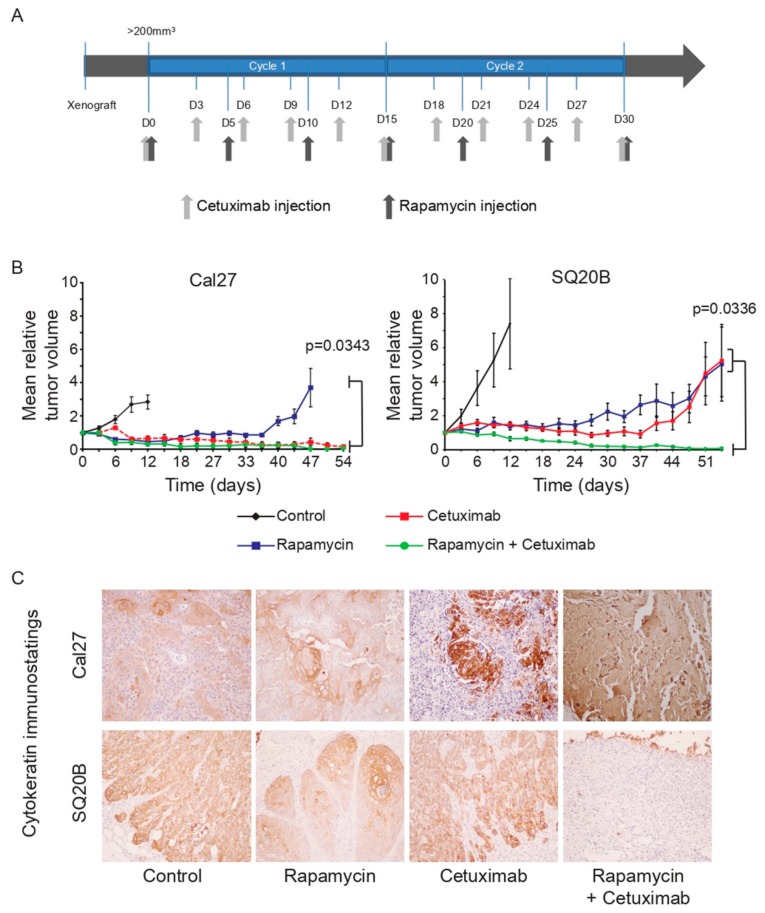
Effect of cetuximab and rapamycin treatment on SQ20B and Cal27 xenografts. (**A**) Treatment schedule of nude mice bearing Cal27 and SQ20B xenografts. (**B**) Mean relative tumor volume of Cal27 and SQ20B xenografts measured during and after treatment (*n* = 10 tumors per group). Error bars represent the standard error in each panel. Statistical significance was evaluated after the completion of the 2 treatment cycles. Bracket show statistically significant differences (Kruskal–Wallis p-values are shown). (**C**) Immunohistochemistry analysis of hematoxylin and pan-cytokeratin staining in xenograft tissue harvested from nude mice after the completion of the treatment. One representative micrograph is shown for each treatment arm for both cell lines. Pan-cytokeratin staining is visible in brown. Hematoxylin blue staining was used to counter-color the whole tissue. Please note: residual post-treatment Cal27 xenografts show no positive hematoxylin nuclei and display non-specific brown staining of necrotic tissue. Magnification: 20×.

To assess the in vivo efficacy of these regimens, nude mice bearing Cal27 or SQ20B cell lines-derived heterotopic xenografts were randomized to treatment arms consisting of two cycles of injections of rapamycin (3 mg/kg; q7d5, i.e., total of 7 injections with 1 injection every 5 days; Figure 1A) and cetuximab (1 mg/injection; q11d3, i.e., total of 11 injections with 1 injection every 3 days; Figure 1A), alone or in combination, over 30 days. Rapamycin administration alone resulted in relative tumor volume stabilization but rapid tumor regrowth after the end of treatment in both xenograft models (Figure 1B). Consistently, post-treatment tumors stained with standard hematoxylin/eosin and pan-cytokeratin immunohistochemistry displayed remaining carcinoma cells (Figure 1C; Figure S2A). Contrastingly, cetuximab alone induced long-term tumor shrinkage of Cal27 xenografts, early in treatment (Figure 1B). Pan-cytokeratin staining of residual tissue dissected from nude mice after treatment showed a strong diminution of remaining carcinoma cells in the samples that were examined (Figure 1C; Figure S2A, Table 1), suggesting that the cetuximab has an anti-tumor effect on Cal27 xenografts. However, SQ20B tumors exhibited intermediate sensitivity to anti-EGFR therapy, with accelerated regrowth at the end of treatment (positively stained carcinoma cells remained after treatment; Figure 1B,C; Figure S2A). Strikingly, the addition of rapamycin significantly enhanced the efficacy of cetuximab in the SQ20B xenografts, resulting in tumor shrinkage without relapse up to 6 months post-treatment (Figure 1B,C; Figure S2A). Impairment in HIF-1α expression and Carbonic Anhydrase IX (CAIX) was assessed using fluorescent immunohistochemistry (Figure S2B). Indeed, the gene that encodes CAIX is a direct transcriptional target of HIF-1α, and expression of CAIX protein was therefore used as a molecular read-out for HIF-1α transcriptional activity. Cal27 xenografts from naïve or treated mice showed no positive HIF-1α or CAIX staining, suggesting an absence of hypoxic regions in these tumors. However, carcinoma cells with nuclear HIF-1α or membranous CAIX staining could be observed in SQ20B xenografts harvested from non-treated mice. Administration of cetuximab alone did not impact these expression patterns whereas treatment with rapamycin or with rapamycin and cetuximab resulted in a significant reduction of both HIF-1α and CAIX staining. Altogether, these results show that the pharmacological inhibition of the EGFR/mTOR/HIF-1 axis has an effective anti-tumor activity in a resistant model of HNSCC cell line, in vitro and in vivo.

### 2.2. Targeting the EGFR/mTOR/HIF-1 Pathway Partially Increases the Sensitivity of SQ20B Cells to Ionizing Radiation

**Figure 2:**
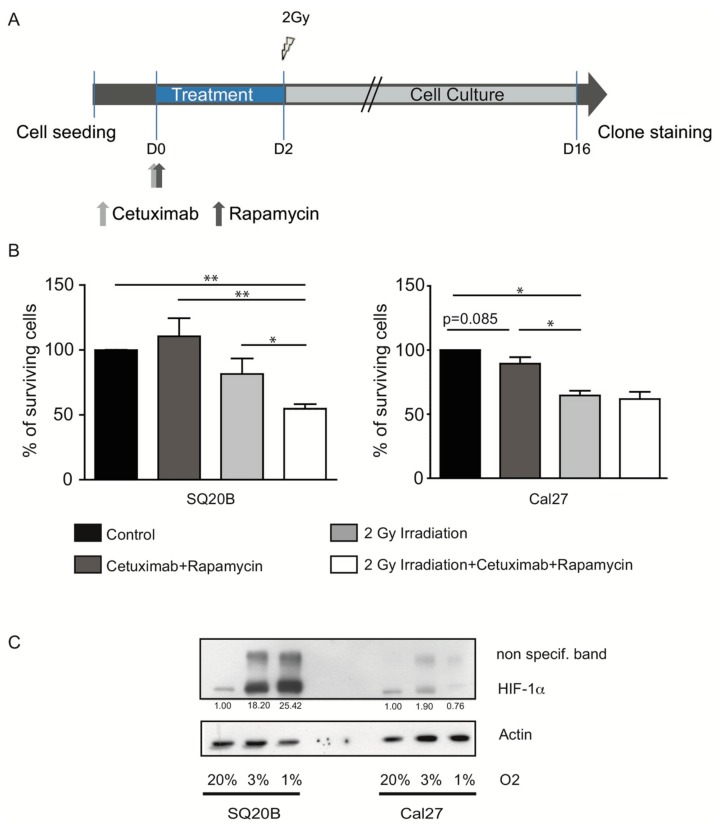
Epidermal Growth Factor Receptor (EGFR)/mTOR axis inhibition sensitizes SQ20B radioresistant cells. (**A**) In vitro treatment schedule of Cal27 and SQ20B cells. (**B**) Clonogenic survival assay of SQ20B and Cal27 cells after cetuximab/rapamycin treatment and 2Gy irradiation, delivered alone or in combination. Results from at least 3 independent experiments are shown. Error bars represent the standard deviation. (Kruskal–Wallis test and two-side Mann–Whitney: test; * *p* < 0.05; ** *p* < 0.01). (**C**) Hypoxia-Inducible Factor-1 (HIF-1α) expression at the protein level in SQ20B and Cal27 cell lines cultured in normoxic (20% O2) and hypoxic (3% and 1% O2) conditions. Signal quantifications (normalized to actin levels for each condition and expression level in normoxic conditions set to a value of 1) are shown.

We then evaluated the radiosensitizing effect of the drug combination in vitro. Since HIF-1 has been implicated in radioresistance, we figured that effective EGFR/mTOR/HIF-1 axis inhibition could improve the impact of subsequent radiation. Therefore, cells were treated with 2.5 µg/mL cetuximab, 5 nM rapamycin, for 48 h, and further exposed to 2 Gy of γ-rays (Figure 2A). The surviving fraction of cells with colony-formation abilities was assessed in a clonogenic assay. As expected, the clonogenic survival of Cal27 significantly dropped to ~65% upon irradiation, but inhibition of the EGFR/mTOR/HIF-1 axis with rapamycin and cetuximab before radiation therapy had no further specific effect. In contrast, a ~45% drop in survival of SQ20B cells was observed after treating cells with both irradiation and the rapamycin/cetuximab combination (two-side Mann–Whitney test; * *p* < 0.05; Figure 2B). This result correlated with a lower expression of HIF-1α in Cal27 as compared to SQ20B cell line in untreated conditions (Figure 2C).

Finally, the generation of DNA double strand breaks (DSBs) was assessed in SQ20B cells using γH2AX staining (Figure S3A,B). γH2AX foci were significantly increased when cells were treated with the cetuximab/rapamycin combination before irradiation, suggesting that this regimen could radiosensitize SQ20B cells by DNA breaks accumulation.

### 2.3. EGFR Inhibition and Ionizing Radiation Induce HIF-2α Expression in SQ20B Cells

**Figure 3:**
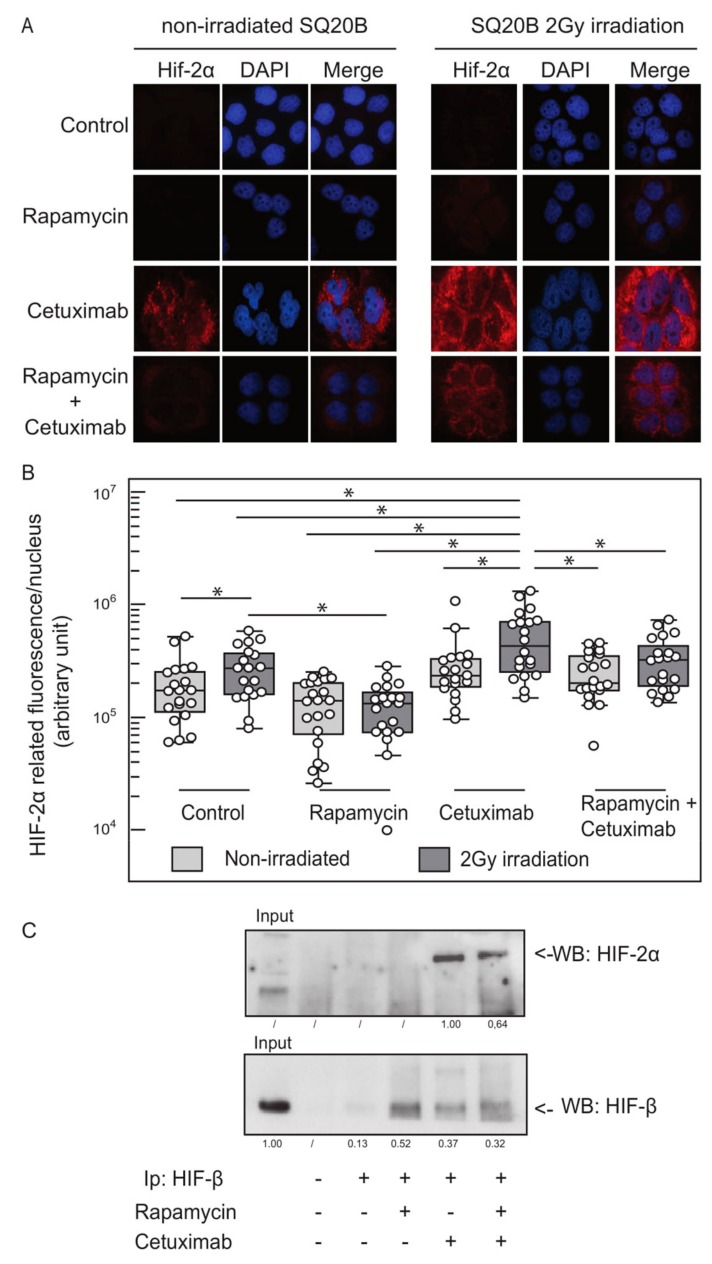
Cetuximab and ionizing radiation induce HIF-2α expression in SQ20B cells. (**A**) Fluorescent immunohistochemistry analysis of HIF-2α staining (red) performed on SQ20B cells treated with cetuximab and/or rapamycin, and 2Gy irradiation delivered alone or in combination. Cell nuclei are stained with DAPI (4′,6-diamidino-2-phénylindole; blue). Three biologically independent experiments were performed, and representative micrographs are shown. (**B**) Quantitative analysis of HIF-2α immunohistochemical staining shown in Figure 4A. Quantification was performed on 10 independent fields from 3 independent experiments. Results are plotted as a box-and-whisker plot representing the median value, the 25th and 75th quartiles. Data in different populations was compared using a Kruskal–Wallis test and two-side Mann-Whitney post-test; * *p* < 0.05). (**C**) Analysis of HIF-2α expression in SQ20B cells after cetuximab and rapamycin treatments, alone or in combination. Protein extracts were immunoprecipitated with an anti-HIF-β antibody. Purified proteins were resolved by sodium dodecyl sulphate-polyacrylamide gel electrophoresis (SDS-PAGE) and membranes were probed with an anti-HIF-2α antibody or an anti-HIF-β antibody (control). Signal corresponding to HIF-2α (upper panel) and HIF-β proteins (lower panel) are highlighted with an arrow. Signal quantifications are shown: HIF-2α signal was normalized to signal in SQ20B cells treated with cetuximab, which was set to 1; HIF-β signals were normalized to the input, which was set to 1; signal was not detected in certain conditions which are labeled as/.

Although the combination of cetuximab and rapamycin treatment with radiation therapy was relatively effective in vitro, it failed to fully eliminate carcinoma cells in the clonogenic assays. HIF-1α and HIF-2α are homologous factors that both interact with HIF-β to form the HIF-1 and HIF-2 heterodimeric transcription factors, respectively. Both factors are induced upon low oxygen pressure and play a role in the cellular response to hypoxia by binding to hypoxia-responsive elements and regulating the expression of common and specific target genes [14,15]. Therefore, we hypothesized that HIF-1α inhibition obtained after cetuximab and rapamycin exposure could functionally be compensated for by the induction of HIF-2α. HIF-2α expression was, therefore, monitored at the RNA and protein levels in naive and treated cells, by using quantitative reverse transcription polymerase chain reaction (qRT-PCR) and Western blots approaches, respectively. We observed that cetuximab or ionizing radiation induced a 3- to 4-fold increase of HIF-2α mRNA (data not shown). Accordingly, immunofluorescent analysis showed a striking induction of the HIF-2α protein in SQ20B cells grown in the presence of cetuximab, and this effect was further increased by ionizing radiation (Figure 3A,B). Interestingly, incubation of cells with rapamycin impaired HIF-2α expression to a certain extent in irradiated cells. The induction of HIF-2α expression upon cetuximab treatment and the presence of HIF-β/HIF-2α heretodimeric transcription factors was further validated by a Western blot analysis of SQ20B cell whole protein extracts where HIF-2α was enriched by immunoprecipitation with an anti-HIFβ antibody (Figure 3C).

Finally, we wanted to evaluate if HIF-2α induction was specific to cetuximab treatment, or could be induced upon EGFR inhibition using other pharmaceutical agents. In order to address this question, SQ20B cells were, therefore, exposed to two ATP-competitive tyrosine kinase inhibitors, erlotinib (5 μM) or canertinib (5 μM) alone, and results were compared to cetuximab. HIF-2α expression was analyzed by immunofluorescence and results showed a significant increase in the protein accumulation with both treatments (Figure S4).

### 2.4. Basal and Cetuximab-Induced HIF-2α Expression Levels Correlate with Resistance to EGFR Inhibition

**Figure 4:**
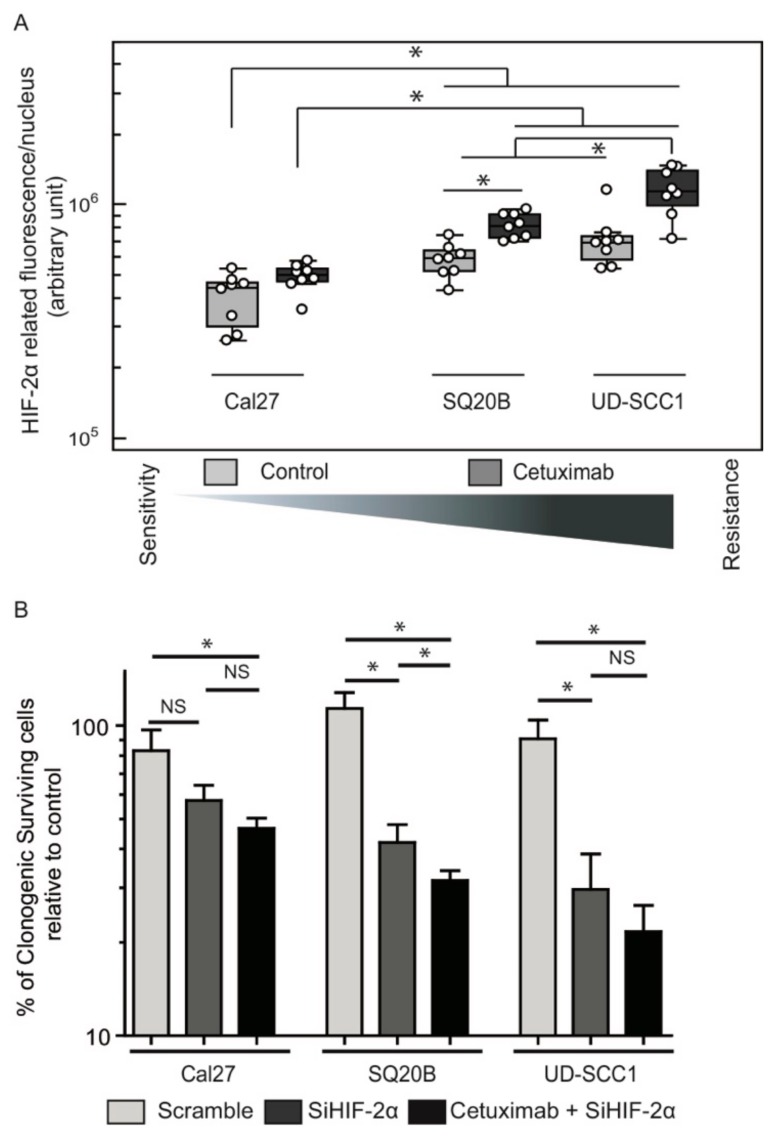
Cetuximab-induced HIF-2α expression results in oncogenic addiction. (**A**) Relative HIF-2α protein expression evaluated by fluorescent immunohistochemistry on Cal27, SQ20B and UD-SCC1 cells. Quantification of the signal in each experimental condition was performed on 8 to 10 independent fields from 2 independent experiments (i.e., 4 to 5 fields per experiment). Results are plotted as a box-and-whisker plot representing the median value, the 25th and 75th quartiles. Data in different populations was compared using a Kruskal-Wallis test (data distribution is non-normal) and two-side Mann–Whitney post-test; * *p* < 0.05. (**B**) Clonogenic survival assay of Cal27, SQ20B and UD-SCC1 cells treated with cetuximab and/or transfected with anti-HIF-2α siRNA from 3 independent experiments. Error bars represent the standard deviation (Kruskal–Wallis test and two-side Mann–Whitney test; * *p* < 0.05).

We analyzed the functional relationship between HIF-2α expression and the response to EGFR inhibition. To this end, we measured the expression of HIF-2α in naive cells and cells that were treated with cetuximab. This experiment was carried out on cetuximab-sensitive (Cal27) and cetuximab-resistant (SQ20B; UD-SCC1) cells. Cal27 cells displayed low basal HIF-2α expression levels, and no significant increase of HIF-2α expression was observed upon cetuximab treatment (Figure 4A). However, HIF-2α expression was found to be higher and upregulated by cetuximab in SQ20B and UD-SCC1 (Figure 4A). These observations suggest a correlation between the cell response to cetuximab and the endogenous and/or induced expression of HIF-2α.

This hypothesis was functionally challenged by silencing HIF-2α expression with a siRNA approach. A specific anti-HIF-2α siRNA was used, which achieved a >80% inhibition of both transcript and protein expression in each cell line (Figure S5A) and protein (Figure S5B) expression inhibition in each cell line compared to the scrambled siRNA. The inhibition of HIF-2α expression in Cal27 cells resulted in a moderate, non-significant drop in clonogenic survival (83% vs. 57%; Figure 4B). In contrast, the inhibition of HIF-2α resulted in a significant decrease of clonogenic survival of SQ20B (100% vs. 42%, *p* = 0.038) and UD-SCC1 (91% vs. 30%, *p* = 0.05) cells compared to transfection with the scrambled siRNA (Figure 4B). Treatment of SQ20B and UD-SCC1 cells with cetuximab further reduced clonogenic survival to 32% and 22%, respectively, although the difference did not reach statistical significance in UD-SCC1 cells (Figure 4B).

**Figure 5:**
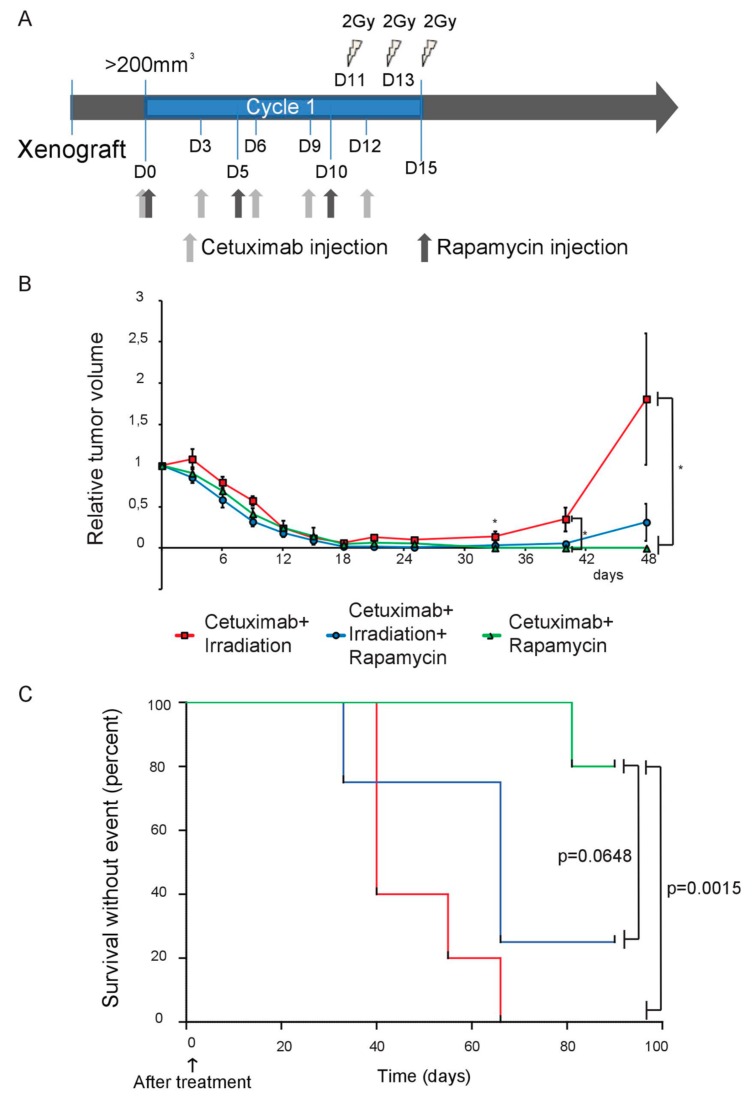
Radiation therapy accelerates tumor relapse of resistant SQ20B xenografts. (**A**) Treatment schedule of nude mice bearing SQ20B xenografts. (**B**) Mean relative tumor volume of SQ20B xenografts measured during and after treatment (*n* = 10 tumors per group). Error bars represent the standard deviation in each panel. Statistical significance was evaluated after the completion of the 2 treatment cycles (Kruskal–Wallis test and two-side Mann–Whitney test; * *p* < 0.05). (**C**) Kaplan–Meier survival curves of mice carrying SQ20B xenografts. A log-rank test analysis showed that mice treated with the cetuximab/rapamycin combination (green curve) displayed a close-to-significantly better survival than mice treated with cetuximab/rapamycin/radiotherapy (blue curve; *p* = 0.0648), and a significantly better survival than mice treated with cetuximab/radiotherapy (red curve; *p* = 0.0015).

In order to further confirm these observations in vivo using a clinically relevant therapeutic strategy, we evaluated the impact of radiotherapy provided together with cetuximab/rapamycin combination. However, since two cycles of this regimen resulted in complete tumor shrinkage (Figure 1A,B), we decided to apply only one cycle of cetuximab and rapamycin. In addition, and similarly to our in vitro experiment settings, we pre-inhibited the EGFR/mTOR/HIF-1 axis before applying ionizing radiation. To this end, mice bearing SQ20B heterotypic xenografts were randomized in treatment arms (Figure 5A) and received fractionated radiotherapy (6 Gy, delivered in 3 daily doses) on days D11, D13 and D15. Cetuximab was delivered from D0 to D12 alone (1 mg/injection; q5d3, i.e., total of 5 injections with 1 injection every 3 days), or in combination with rapamycin (3 mg/kg, q3d5, i.e., total of 3 injections with 1 injection every 5 days). All treatments achieved effective tumor shrinkage (Figure 5B). Consistent with our previous observation, delivery of 1 cycle of cetuximab and rapamycin combination achieved 90% control rate of tumor regrowth after the end of treatment (1 out of 10 tumor relapsed 45 days post-treatment; Table 2, Figure 5B). However, the concomitant delivery of radiation therapy with this combination had a post-treatment adverse effect, with accelerated relapse and reduced control rate (5/8 tumors relapsed 15 days post-treatment; Table 2, Figure 5B). All mice treated with a combination of cetuximab and radiotherapy showed rapid tumor regrowth (10/10 tumors relapsed 3 days after the last irradiation; Table 2, Figure 5B). These relapses had an effect on mice survival (Figure 5C): the cetuximab/rapamycin combination was found to result in longer survival compared to the cetuximab/rapamycin/irradiation (close-to-significant difference: *p* = 0.0648) and to the cetuximab/irradiation (significant difference: *p* = 0.0015) regimens.

## 3. Discussion

Among general resistance mechanisms, chronic/intermittent hypoxia and hypoxia-inducible factors are known to adversely impact tumor response to radiation and chemotherapy in many types of cancer [16,17,18,19], and especially in HNSCC [10,20] where hypoxia and HIF-1 have been shown to induce the upregulation of EGFR [8]. In addition, HIF-1α expression can be regulated independently of oxygen pressure by oncogenic pathways, including the EGFR/PI3K/mTOR pathway [13]. Since high EGFR expression is a negative prognostic factor, associated with lower progression-free and overall survival rates [21], we reasoned that resistant head and neck cancer cells could be re-sensitized to ionizing radiation by targeting EGFR and mTOR with cetuximab and rapamycin, respectively. The radiosensitivity of HNSCC cells was previously shown to improve upon impairing of HIF-1α expression [22] and the mTOR inhibitor CCI-779 was shown to potentiate the efficacy of radiotherapy in HNSCC xenograft models [23]. Similarly, treatment of head and neck cancer cell lines with OSI-027, an mTORC1/2 inhibitor, synergistically enhances the effect of the reversible EGFR tyrosine kinase inhibitor erlotinib on cell survival [24]. Agreeing with this data, we found the combination of rapamycin and cetuximab to inhibit HIF-1α more effective than both agents used as monotherapies. As previously demonstrated [25], the rapamycin/cetuximab treatment shows remarkable efficacy in vivo, since nude mice bearing Cal27 xenografts, a cetuximab-sensitive model, showed complete tumor regression and were free of relapse 6 months post-treatment. Interestingly, animals were treated with lower doses of cetuximab and rapamycin in our work, compared to injection protocols traditionally used in literature. In our study, we used SQ20B cells as a cetuximab-resistant model, representing the most frequent phenotype in HNSCC patients. Since locally advanced metastatic HNSCC are managed with concomitant radiotherapy and cetuximab, we evaluated the efficacy of the cetuximab/rapamycin combination provided with ionizing radiation. Interestingly, components of the mTOR pathway are induced by photon radiotherapy and contribute to HIF-1α induction [26], which can be prevented by cetuximab [27]. We hypothesized that cotreatment with rapamycin could further potentiate this effect, and this hypothesis was challenged in nude mice models bearing SQ20B xenografts treated with concomitant radiotherapy and cetuximab or the cetuximab/rapamycin combination. Surprisingly, and although the chemotherapy regimens we used were able to inhibit HIF-1α expression, we observed that all SQ20B xenografts relapsed rapidly after concomitant cetuximab and radiation therapy. This effect was partially contained by the addition of rapamycin. In vitro evaluation of treatment efficacy via measurement of SQ20B clonogenic survival after treatment consistently showed that, although ionizing radiation combined with rapamycin and cetuximab induced increased DNA DSBs and a significant drop of surviving fraction, about 50% of the cells were still able to generate positive clones.

HIFs are heterodimeric transcription factors composed of an oxygen-dependent HIFα subunit and a constitutively expressed HIF-β subunit. Three complexes have been identified, i.e. HIF-1, HIF-2 and HIF-3, but HIF-3 lacks the C-terminal transactivation domain and has multiple splice variants that may act as dominant negative regulators of HIF-1 and HIF-2. HIFs are differentially regulated by hypoxic conditions and HIF-1 has been implicated in acute response to low oxygen tension, whereas HIF-2 activity is maintained during chronic hypoxia [28]. HIF-1 and HIF-2 have overlapping and distinct target genes, depending on the cell type, post-translational modifications or coregulators [14,15]. HIF-2α was previously shown to be expressed in HNSCC [11,29,30] and high HIF-2α expression in HNSCC correlates with increased micro-vessel density, incomplete response to chemotherapy and adverse prognosis [30]. Therefore, we hypothesized that induced expression of HIF-2 upon cetuximab and radiation therapy could functionally compensate the inhibition of HIF-1, and participate to the resistance of HNSCC cells to EGFR blockade. So in order to elucidate the molecular bases of SQ20B cells resistant phenotype, we measured the expression of HIF-2α. We found HIF-2α to be strongly induced in SQ20B cells after exposure to cetuximab, and this effect was further increased by radiation therapy. In addition, when we compared the endogenous and/or cetuximab-induced expression of HIF-2α in three different HNSCC cell lines (Cal27, SQ20B and UD-SCC1), we found a positive correlation between HIF-2α expression levels and resistance to EGFR-inhibition. HIF-2α knock-down using a siRNA approach resulted in a significant reduction of clonogenic survival abilities in cells with high basal/inducible HIF-2α, whereas cells with lower HIF-2α were poorly sensitive to HIF-2α inhibition. Indeed, we observed that the Cal27 cell line that expresses lower endogenous levels of HIF-2α and that does not overexpress HIF-2α upon cetuximab exposure is insensitive to HIF-2α inhibition. By contrast, SQ20B and US-SCC1 cells, which display a higher constitutive and cetuximab-inducible HIF-2α expression, are significantly impacted by HIF-2α inhibition. Interestingly, a similar HIF-2α induction was observed when SQ20B cells were exposed to EGFR blockade by using the tyrosine kinase inhibitors erlotinib and canertinib. These results confirm that HIF-2 therapy-induced expression is probably a more general mechanism implicated in chemo/radioresistance in several solid tumor models. For example, in non-small cell lung cancer cell lines, HIF-2α promotes the MET proto-oncogene expression and induces resistance to Gefitinib and Erlotinib [31]. Similarly, Sorafenib treatment of hepatocellular cancer cell inhibits HIF-1α but triggers HIF-2α expression and resistance to the treatment [32,33].

A growing body of experimental evidence suggests that cancer initiating/stem cells are involved in resistance to treatments [34] and that hypoxia is a key contributor to stemness promotion or maintenance [35,36]. Among hypoxia inducible factors, HIF-2 has been shown to positively regulate the transcription of pluripotency genes such as NANOG, SOX2 and OCT4 [37]. In addition, HIF-2 activates stemness via Wnt and Notch signaling, and induces treatment resistance in vitro and in vivo [10,38].

## 4. Materials and Methods

### 4.1. HNSCC Cell Lines Treatments and Proliferation Assay

Human head and neck squamous carcinoma SQ20B [39], Cal27 [40], and UD-SCC1 [41] cell lines used in this study were generated from laryngeal, tongue and oropharyngeal tumors, respectively. Cell lines were maintained at 37 °C in normoxic (21% O_2_, 5% CO_2_) or hypoxic (94% N_2_, 5% CO_2_, 1% O_2_) conditions in Dulbecco’s Modified Eagle Medium (DMEM; 1 g/L glucose) supplemented with 10% fetal bovine serum. SQ20B cells were a kind gift of Dr Pierre Bischoff; Cal27 cells were a kind gift of Dr Sophie Pinel; UD-SCC1 were a kind gift of Pr Thomas K Hoffmann. SQ20B, UD-SCC1 and Cal27 cell lines authentication and/or absence of cross-contamination were last performed by DNA (Short Tandem Repeats) profiling (16 loci) on genomic DNA in August 2016 and verified in November 2017. Cells were treated during exponential growth conditions (70% confluence); 3000 cells per well were seeded in 96-well flat-bottomed plates and incubated for 48 h with cetuximab (0.25 to 50 μg/mL), rapamycin (0.1 to 500 nM), or a combination of both drugs (5 nM rapamycin and 0.25 to 50 µg/mL Cetuximab). For irradiation experiments, cells were plated 24 to 48 h prior to irradiation, and a single 2 Gy γ-ray irradiation dose was delivered with a 137Cs γ-irradiator (Biobeam 8000 irradiator, Gamma Service Medical, Leipzig, Germany) at the Centre Paul Strauss irradiation facility. Concentration (5 µM) of Erlotinib and Canertinib used for in vitro analysis of HIF-2 induction was chosen based on the literature and clinical practice. Both molecules have a similar IC50 on isolated EGFR (0.3–1.7 nM) [42], and in patients, Cmax (plasma) obtained after daily 150 or 300 mg oral dose of Erlotinib ranges approximatively from 2.5 µM to 5.0 µM (1000 to 2000 ng/mL) [43]. In addition, most HNSCC cell lines show a narrow range of in vitro sensitivity to Erlotinib (about 2–6 μM; see [44,45]).

Cell proliferation was measured using a Sulforhodamine B proliferation assay (Sigma Aldrich, Lyon, France) and coloration was quantified using a Synergy HT^®^ Biotek plate reader according to the manufacturer’s instructions.

### 4.2. Animals and Tumor Xenografts

The animal study was approved by the French Ethical Committee (AL/86/93/02/13 12/11/29) and performed under the supervision of authorized investigators. Female athymic nude mice (nu/nu), 6 to 8 weeks old, were purchased from Charles River and maintained under pathogen-free conditions. Cal27 and SQ20 xenografts were obtained by injecting cell suspensions subcutaneously into the flank of mice. Two tumors per mouse were generated (one on each flank). Treatments were delivered to nude mice by intraperitoneal injections (i.p) when tumors reached an average volume of 150–200 mm^3^ and mice were randomized into 4 groups: control (non-treated mice); cetuximab (1mg every 3 days); rapamycin (3 mg/kg every 5 days); combination of cetuximab and rapamycin at the same doses and according to the same schedule (Figure 1A). Cetuximab and rapamycin concentrations were set according to U.S. FDA guidelines (Guidance for industry. Estimating the maximum safe starting dose in initial clinical trials for therapeutics in adult healthy volunteer; https://www.fda.gov/media/72309/download; see also [46]). 1 mg cetuximab every 3 days corresponds to cetuximab administration in humans in the EXTREM regimen (i.e., 250 mg/m^2^ weekly); 3 mg/kg rapamacyn corresponds to the usual Rapamune® posology of 1.8 mg/m^2^ for patients undergoing organ transplantation. The treatment was continued for 4 weeks (two cycles of treatment) or 2 weeks (one cycle of treatment). Mice were observed daily for tumor growth, the mean tumor volume (MTV) was measured every 3 days using a caliper and the relative tumor volume (RTV) was calculated for each treatment groups as follows: tumor volume (*V*) was evaluated every 3 days and was calculated as *V* = (*a*^2^ × *b*)/2, where *a* is the width of the tumor in millimeters and *b* is the length in milimeters. The individual relative tumor volume (RTV) was defined as *V*x/*V*0, where *V*x is the volume in mm^3^ at a given time and *V*0 is the volume at the start of treatment. Mean RTV (MTV) and standard error of the mean (SE) were calculated for each group. Tumor irradiation was carried out by protecting mice with lead shield and exposing xenografts only to ionizing radiation on the Centre Paul Strauss irradiation facility, using a 137Cs γ-Biobeam 8000 irradiator (Gamma Service Medical, Leipzig, Germany). Three doses of 2 Gy (Gray) were delivered on day 11, 13 and 15. When cetuximab +/− rapamycin treatment was combined to radiation therapy, irradiation was performed 2 days after the fourth injection of cetuximab. Tumor growth curves were stopped when the first tumor reached a volume >2000 mm^3^, and for survival studies, mice were sacrificed when tumors reached volumes >2000 mm^3^. 

### 4.3. Histologic and Immunohistochemical Studies

One tumor was harvested in each group at midcourse and after completion of the treatment. Tissue fragments were either fixed with a zinc fixative solution and embedded in paraffin for immunohistochemical (IHC) analysis, or rapidly frozen on dry ice, and stored at −80°C. For conventional histology, 4 μm paraffin-embedded sections were stained with hematoxylineosin. For IHC staining of pan-cytokeratin, slides from formalin-fixed paraffin-embedded xenografts were stained with a primary rabbit anti-pan-cytokeratin polyclonal antibody (1/100; Invitrogen, Illkirch, France) and a secondary horseradish peroxidase-conjugated anti-rabbit antibody (1/500; Roche Molecular Biochemicals, Meylan, France). Signal detection was carried out with a DAB substrate kit (Roche diagnostics), according to the manufacturer’s instructions.

### 4.4. Small Interfering RNA Transient Transfection

Two validated stealth small interfering RNAs (Qiagen, Courtaboeuf, France) targeting HIF-2α were used alone or in combination. Cells were transfected using Lipofectamine RNAiMAX, according to the manufacturer’s instructions. 3 × 10^5^ cells were incubated with siRNAs, either alone or in combination, and total protein and RNA extractions were carried out 48 h after transfection.

### 4.5. Clonogenic Survival Assay

Cells were diluted and seeded 24 h after irradiation (i.e., 48 h after cetuximab +/− rapamycin treatment) and incubated in normoxic conditions for up to 2 weeks after irradiation. Clones were stained with crystal violet 0.05% (Sigma Aldrich, Lyon, France) in a 5% ethanol solution, and positive colonies (>64 cells) were counted. The surviving fraction at 2 Gy (SF 2 Gy) was calculated by dividing the number of positive colonies by the number of cells that were seeded, multiplied by the plating efficiency. The plating efficiency was calculated by dividing the number of positive colonies that grew in the absence of irradiation divided by the number of cells that were seeded.

### 4.6. Immunofluorescence Staining of γH2AX, HIF-1, HIF-2, CA-IX

Immunofluorescence staining experiments were performed both on 4 µm tissue sections from paraffin embedded xenografts and Labtek chamber slides. Slides were stained with anti-γH2AX (1/500e; Clone JBW301 Millipore, Molsheim, France), anti-HIF-1α (1/75e; Novus Biologicals, Rennes, France), anti-HIF-2α (1/500; Abcam), and anti-CA-IX (1/500e; Abcam) antibodies. Nuclei were stained with a DAPI solution (1/30,000) and slides were mounted with Aqua Polymount (Polysciences Inc., Le Perray-en-Yvelines, France) Fluorescence images were captured using a fluorescence microscope axioimager Z2 (Zeiss Industries, Marly-Le-Rois, France). All images were acquired using the same variables (exposure time, contrast, and brightness), and were further uniformly processed with Adobe Photoshop software (Adobe Systems, San José, CA, USA) in order to improve the contrast. γH2AX and HIF-2α signal quantification was performed by using the ImageJ software, and normalized to the number of nuclei labeled with DAPI.

### 4.7. Western Blot Analysis

Total protein extraction was carried out by homogenizing 10^6^ cells in 200 µL lysis buffer (10 mmoL/L Tris (pH 7.5), 5 mmoL/L MgCl_2_, 10 mmoL/L NaCl, 0.5% NP-40, protease and phosphatase inhibitors, Sigma); 20 μg of total proteins were resolved by SDS-PAGE according to standard methods. Proteins were detected with anti-HIF-1α (1/500e; BD Biosciences, Allschwill, Switzerland), anti-HIF-2α (1/500; Abcam), polyclonal anti-α/β-tubulin (1/2000; Cell Signaling, Leiden, The Netherlands), polyclonal anti-actin (1/20000; Santa Cruz Biotech, Heidelberg, Germany), anti-S6 ribosomal protein (1/1000; Cell Signaling) and anti-phospho-S6 ribosomal protein (1/1000; Cell Signaling, Leiden, The Netherlands).

### 4.8. Immunoprecipitation

We diluted 5 µg of nuclear protein extracts to a final volume of 1 mL with immunoprecipitation buffer (150 mM NaCl, 50 mM TrisHCl (pH 8), 1% NP40, complete Protease Inhibitor Cocktail (Roche ref 11697498001) and incubated with a mouse anti-HIF1β antibody (Abcam 2771). Immunoprecipitation was carried out by using protein G-agarose beads. Immunoprecipitated proteins were resuspended in Laemmli buffer after standard centrifugation, and quantified by Western blot analysis with rabbit anti-HIF2-α analysis (1/500, Abcam).

### 4.9. Statistical Analysis

Data analysis of results was performed from with GraphPad Prism software version 5.2. The non-parametric Kruskal–Wallis rank test was used to simultaneously compare non-normally distributed quantitative variables for more than 2 groups. Further bilateral comparison of 2 groups of quantitative variables with non-normal distributions were made using the non-parametric Mann–Whitney post hoc test. A significant *p*-value on a Kruskal–Wallis test means that the mean of at least one group of variables is statistically different from the mean of other groups. The Mann–Whitney post hoc test further analyzes each group of variables with respect to the others. Differences between groups (tumor size, proliferation, clonogenic survival fraction) were considered statistically significant for *p* values < 0.05. Survival curves analysis was performed with the Kaplan–Meier method and significance was analyzed with the log rank test.

## 5. Conclusions

Overall, growing evidence support that high expression of HIF-2α, whether endogenous or induced upon treatment, could be responsible for treatment failure, and underline the urgent need for the development of HIF-2 targeted therapies. A further dissection of both the molecular mechanisms that underlie the induction of HIF-2α upon treatment and the cellular read-outs regulated by HIF-2 are required to improve our knowledge of how cell resistance is triggered by HIF-2 in order to identify the most promising anti-HIF-2 therapies. Therefore, HIF-2 is an attractive therapeutic target and combining HIF-2 inhibitors to conventional or targeted treatments may prove to be useful clinically to delay or prevent resistance.

## Figures and Tables

**Table 1:** Cetuximab and rapamycin co-treatment prevents tumor relapse in nude mice bearing SQ20B xenografts. Nude mice bearing SQ20B and treated with 2 cycles of rapamycin or cetuximab (see Figure 1A for treatment schedule) all show immediate tumor progression upon the cessation of treatment. A cetuximab + rapamycin co-treatment prevented tumor relapse in all mice for up to 6 months after treatment. The number of mice that were treated, and the percentage of tumors that relapsed after treatments, as well as the time to progression are shown. NA (not applicable): number of tumor regrowth, regrowth incidence and time to progression were not evaluated because corresponding treatment only stabilized tumor volume without inducing lesion shrinkage.

	Treatment Arm	# Mice	# Tumors	Regrowth (# Tumors)	Regrowth Incidence	Time to Progression
**SQ20B** **Xenograft**	Non-treated	5	10	10	100%	NA
	Rapamycin (2 cycles)	5	10	10	100%	NA
	Cetuximab (2 cycles)	5	10	10	100%	NA
	Cetuximab+Rapamycin (2 cycles)	5	10	0	0%	None (6 months follow-up)

**Table 2:** Cetuximab and radiotherapy accelerates tumor relapse in nude mice bearing SQ20B xenografts. Nude mice bearing SQ20B cells were randomized to 3 treatments arms: (i) cetuximab (1 cycle: q5d3) + rapamycin (1 cycle: q3d5); (ii) cetuximab (1 cycle: q5d3) + irradiation (3 × 2Gy) or (iii) cetuximab (1 cycle: q5d3) + rapamycin (1 cycle: q3d5) + irradiation (3 × 2Gy). The number of mice that were treated, the percentage of tumors that relapsed after treatment, as well as the time to progression are shown.

	Treatment Arm	# Mice	# Tumors	Regrowth (# Tumors)	Regrowth Incidence	Time to Progression
**SQ20B** **Xenograft**	Cetuximab (1 cycle) +2 Gy irradiation (D11-D13-D15)	5	10	10/10	100%	3 days
	Cetuximab +Rapamycin (1 cycle) +2 Gy irradiation (D11-D13-D15)	4	8	5/8	62.50%	15 days
	Cetuximab +Rapamycin (1 cycle)	5	10	1/10	10%	45 days

## Supplementary Materials

The following are available online at https://www.mdpi.com/2072-6694/11/10/1607/s1, Figure S1: mTOR inhibition increases cetuximab-induced growth suppression in Cal27 and SQ20B cells. Figure S2: Histochemical analysis of SQ20B and Cal27 xenografted tumors. Figure S3: Cetuximab and rapamycin treatment increases DNA breaks accumulation after γ-ray irradiation in SQ20B rardioresistant cells. Figure S4: Anti-EGFR treatments induce HIF-2α expression in SQ20B cancer cell. Figure S5: Efficient HIF-2α silencing after siRNA transfection in Cal27, UD-SCC1 and SQ20B cells. Table S1: Combination index of HNSCC cell lines.
