# Supplementary material for: Constitutive or Induced HIF-2 Addiction is Involved in Resistance to Anti-EGFR Treatment and Radiation Therapy in HNSCC
Source: Cancers (Basel). 2019 Oct 21;11(10):1607. doi: 10.3390/cancers11101607 (PMC6827016; doi:10.3390/cancers11101607)
Supplement: Supplementary file 1 [file cancers-11-01607-s001.zip › Supplementary Materials.docx]

Supplementary Materials

Constitutive or Induced HIF-2 Addiction Is Involved in Resistance to Anti-EGFR Treatment and Radiation Therapy in HNSCC

Pierre Coliat, Ludivine Ramolu, Jérémie Jegu, Christian Gaiddon, Alain C. Jung and Erwan Pencreach

**Figure S1.** mTOR inhibition increases cetuximab-induced growth suppression in Cal27 and SQ20B cells. (**A**,**C**) Cal27 and (**B**,**D**) SQ20B cells were seeded in 96-well flat-bottomed plates and incubated with cetuximab (0.25 to 50 μg/mL), rapamycin (0.1 to 500 nM), or a combination of both drugs (5 nM rapamycin and 0.25 to 50 μg/mL cetuximab) in normoxic (20% O_2_) and hypoxic (1% O_2_) conditions. At 48 h post-incubation, the growth and viability of cells were determined by using a Sulforhodamine B proliferation assay. Results are expressed as percentage of viable cells (mean ± standard deviation (S.D.) of at least triplicate wells) relative to untreated cells. Results from Cal27 (**A**) and SQ20B (**B**) cells treated with increasing concentration of rapamacycin in normoxia and hypoxia are shown in green dotted and red solid lines, respectively. Results from Cal27 (**C**) and SQ20B (**D**) cells grow in hypoxia and treated with 5 nM rapamycin, increasing concentration of cetuximab and increasing concentrations of cetuximab with 5 nM rapamycin are shown as a thin blue dotted straight line, a red solid line and a green dotted line, respectively. (**E**) mTOR/HIF-1α axis inhibition was evaluated by Western blot analysis of Cal27 and SQ20B cells treated by 2.5 μg/mL cetuximab and/or 5 nM rapamycin in hypoxic (1% O_2_) conditions. HIF-1α and pS6RP signal quantifications, normalized to actin or S6RP levels, respectively, for each condition and expression level in non-treated control set to a value of 1 are shown.

**
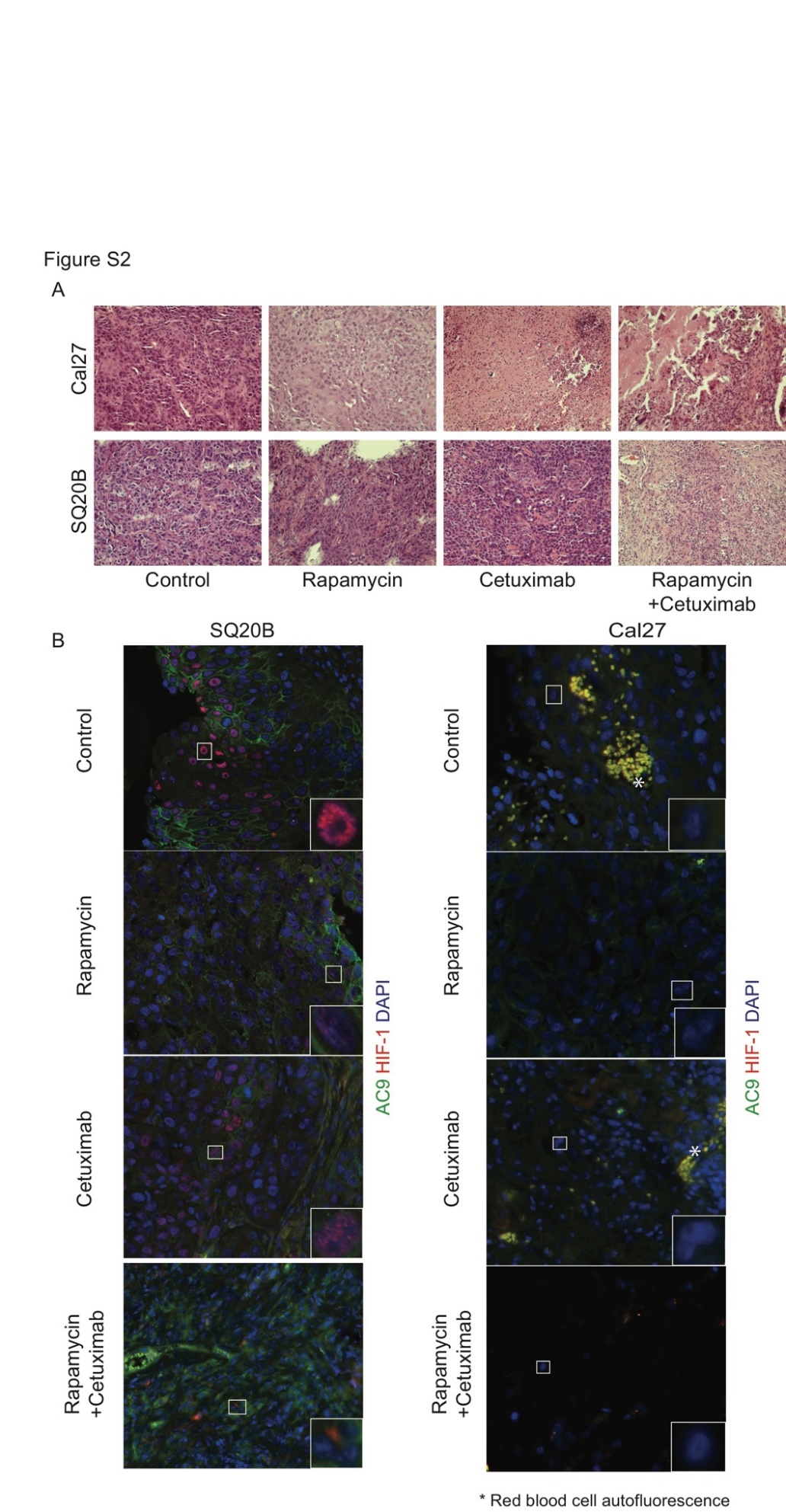
**

**Figure S2.** Histochemical analysis of SQ20B and Cal27 xenografted tumors. (**A**) Hematoxylin-eosin staining of xenografted tumors in untreated or treated conditions (rapamycin and/or cetuximab). (**B**) HIF-1α and CAIX protein expression evaluated analysis by immunofluorescent staining in xenografted tumors samples. Tumors were harvested after completion of the treatment and staining was performed on 4 μm sections from paraffin-embedded tissues. Non-specific erythrocytes auto-fluorescence is visible in yellow (asterisks).


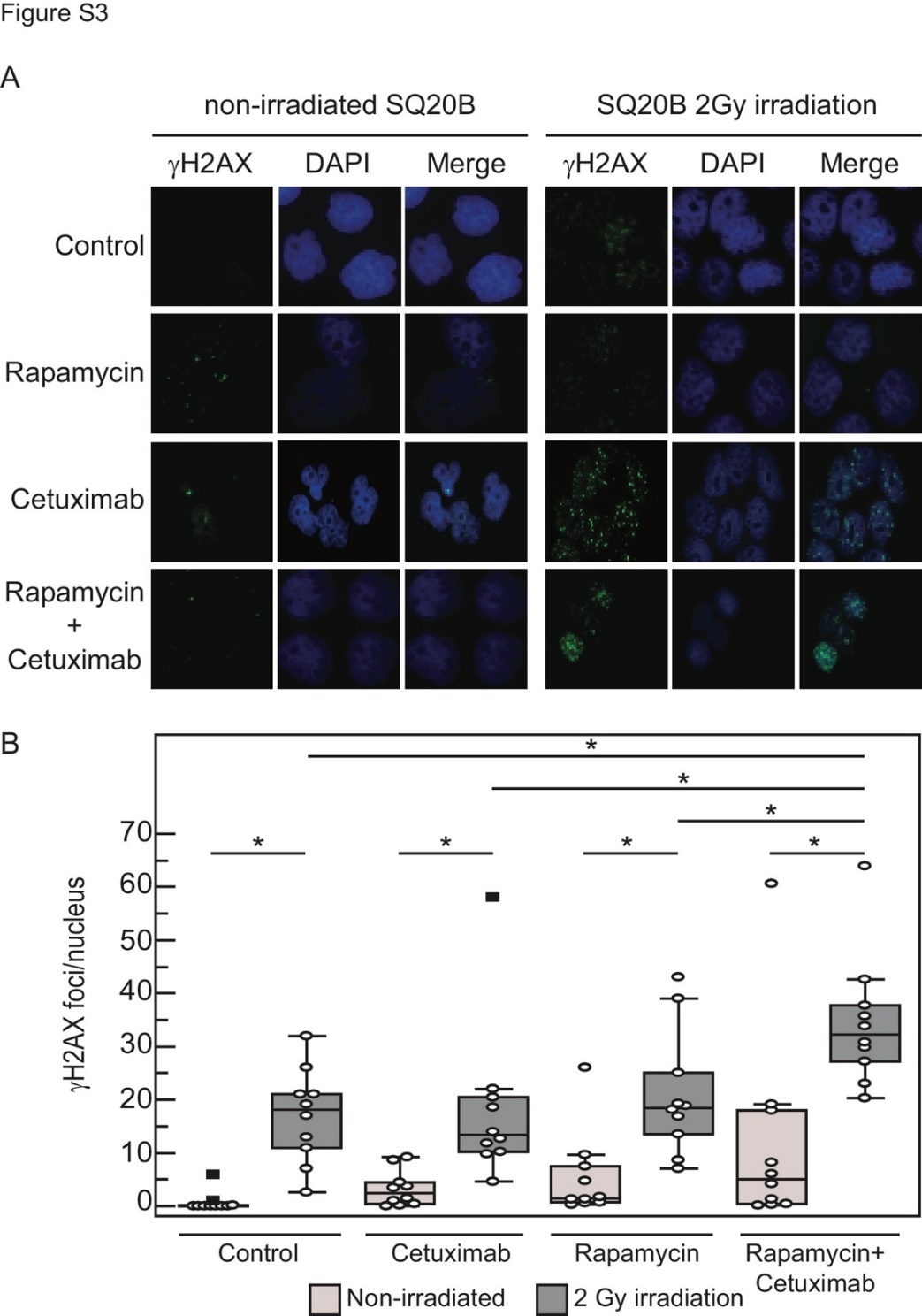


**Figure S3.** Cetuximab and rapamycin treatment increases DNA breaks accumulation after γ-ray irradiation in SQ20B rardioresistant cells. (**A**) Fluorescent immunohistochemistry analysis of γH2AX staining (green) performed on SQ20B cells treated with cetuximab and/or rapamycin, and 2 Gy irradiation delivered alone or in combination. Cell nuclei are stained with DAPI (blue). Two biologically independent experiments were performed, and representative micrographs are shown. (**B**) Quantitative analysis of γH2AX immunohistochemical staining shown in Figure 3A. Quantification was performed on ten independent fields from 3 independent experiments (i.e., 5 fields per experiment). Results are plotted as a box-and-whisker plot representing the median value, the 25th and 75th quartiles. Outliners are represented as black squares. Data in different population was compared using a Kruskal–Wallis test and two-side Mann–Whitney post-test; * *p* < 0.05).


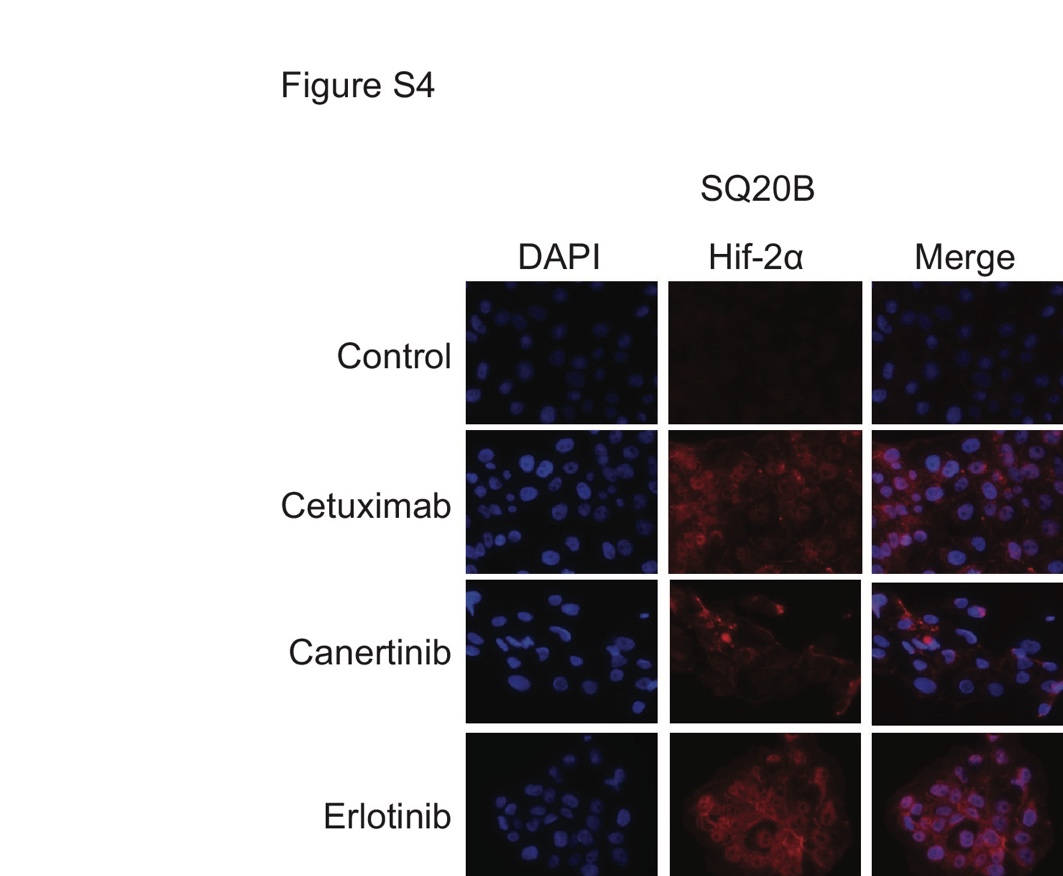


**Figure S4.** Anti-EGFR treatments induce HIF-2α expression in SQ20B cancer cell. Fluorescent immunohistochemistry analysis of HIF-2α expression (red) performed on SQ20B cells, treated with cetuximab, canertinib or erlotinib delivered as monotherapies. Cell nuclei are stained with DAPI (blue). Merge of HIF-2α and DAPI staining is shown for each experimental condition. Representative micrographs from 2 independent experiments are shown.


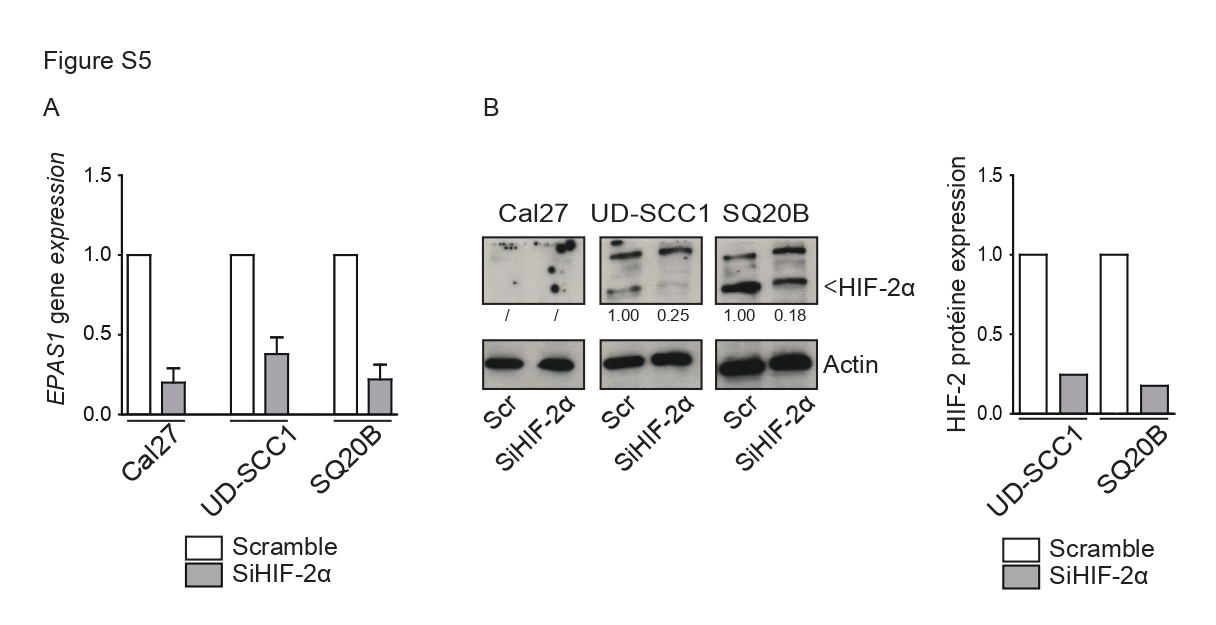


**Figure S5.** Efficient HIF-2α silencing after siRNA transfection in Cal27, UD-SCC1 and SQ20B cells. HIF-2α relative expression was evaluated at the RNA (**A**) and protein (**B**) levels, 48 h after transfection with validated siRNA. Please note that HIF-2α protein was not detected in Cal27 cells. Signal quantifications (normalized to actin levels for each condition and expression level in non-treated control set to a value of 1) are shown. Signal was not detected in Cal27 cells (labeled as/).

**Table S1.** Combination index of HNSCC cell lines. Cal27 and SQ20B cells were treated with 5 nM rapamycin and/or 2.5 µg/mL cetuximab, and anti-proliferative effect of this regimen was measured. Synergism between rapamycin and cetuximab (compared to treatments with drugs alone) was assessed by the method of Chou and Talalay, revised by Fischel and collaborators, and a combination index was calculated for each cell line, in both hypoxic and normoxic cell culture conditions. A combination Index value 0.8 < CI < 1.0 indicates an additive effect.

|  | **CAL27** | | **SQ20B** | |
| --- | --- | --- | --- | --- |
|  | Normoxia | Hypoxia | Normoxia | Hypoxia |
| Combination Index | 0.83 | 0.94 | 0.98 | 0.89 |
| Association | Additive | Additive | Additive | Additive |


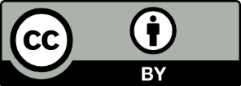
© 2019 by the authors. Licensee MDPI, Basel, Switzerland. This article is an open access article distributed under the terms and conditions of the Creative Commons Attribution (CC BY) license (http://creativecommons.org/licenses/by/4.0/).
