# Supplementary material for: Constitutive or Induced HIF-2 Addiction is Involved in Resistance to Anti-EGFR Treatment and Radiation Therapy in HNSCC
Source: Cancers (Basel). 2019 Oct 21;11(10):1607. doi: 10.3390/cancers11101607 (PMC6827016; doi:10.3390/cancers11101607)

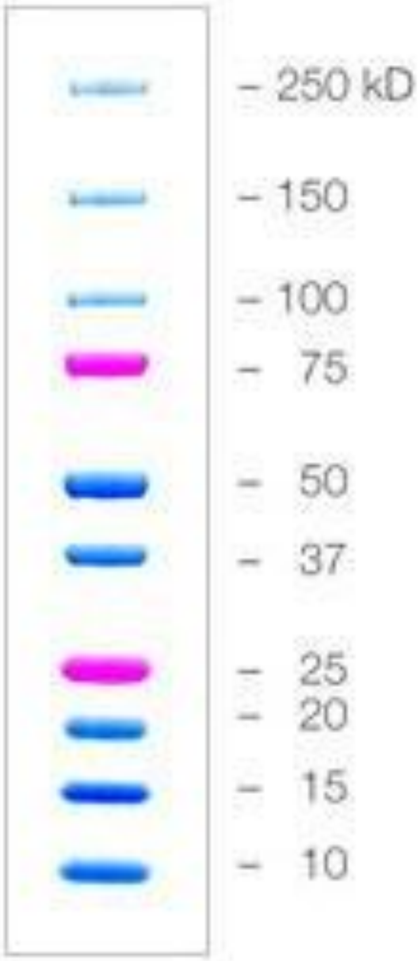

The Biorad dual color protein ladder was used in all western blot analyses (with the exception of the analysis of total S6RP protein)

After protein transfer, membranes were cut under the level of the 75 kD band. Upper parts of the membranes (*i.e.* proteins with molecular weight > 75kD) were incubated with the anti-HIF-1α or anti-HIF-2α or anti-HIFβ antibodies. Lower parts of the membranes (*i.e.* proteins with molecular weight < 75kD) were incubated with anti-actin and anti-pS6RP antobodies.

Above image found at:
https://www.labx.com/item/precision-plus-protein-dual-color-standards-500-181/4215091

Western blots Figure 2C

Blot probed with anti-HIF-1α antibody

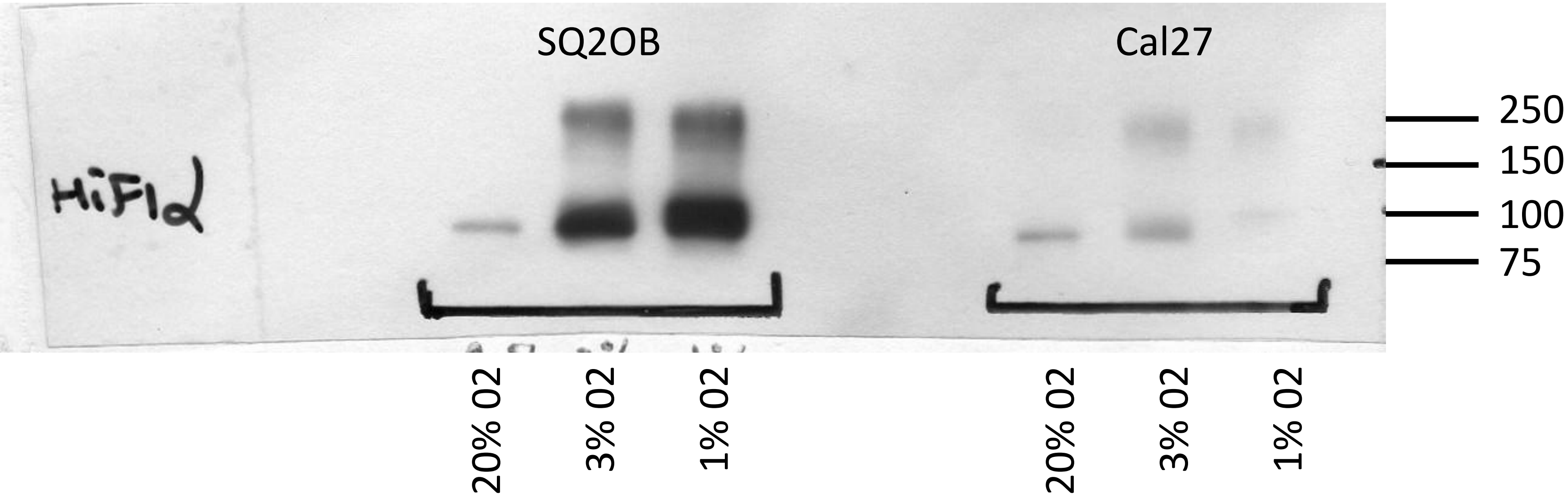

Blot probed with anti-actin antibody

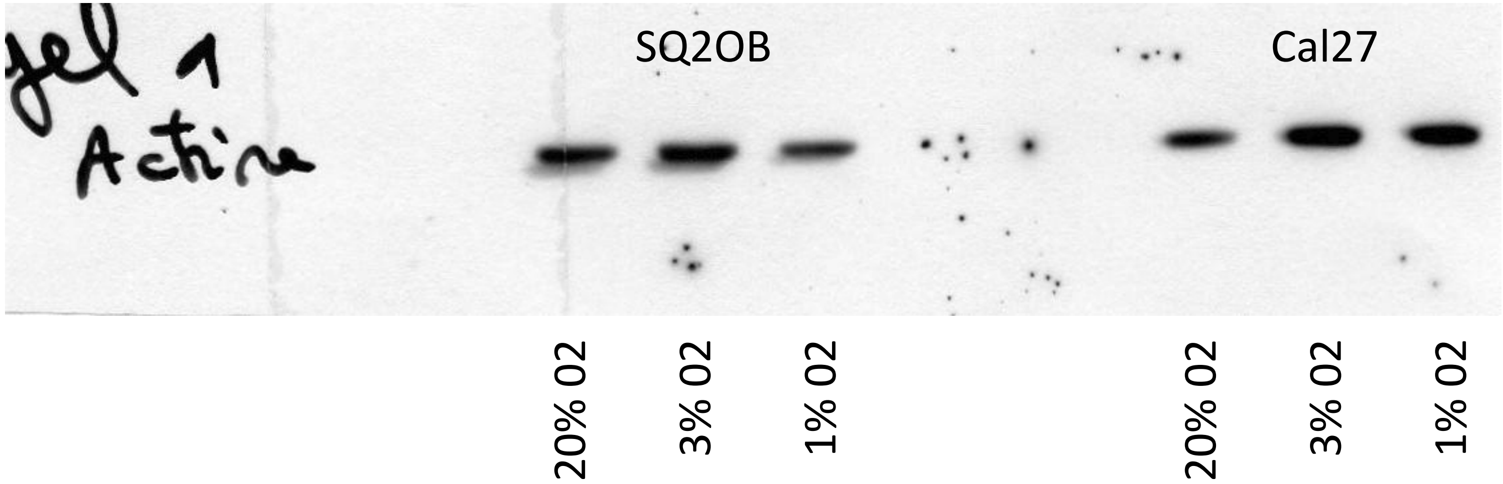

Original blots were cut according to the molecular weight scale and incubated independently with anti-HIF-1α and anti-actin antibodies

Western blots Figure 3C

Blot probed with anti-HIF-2α antibody

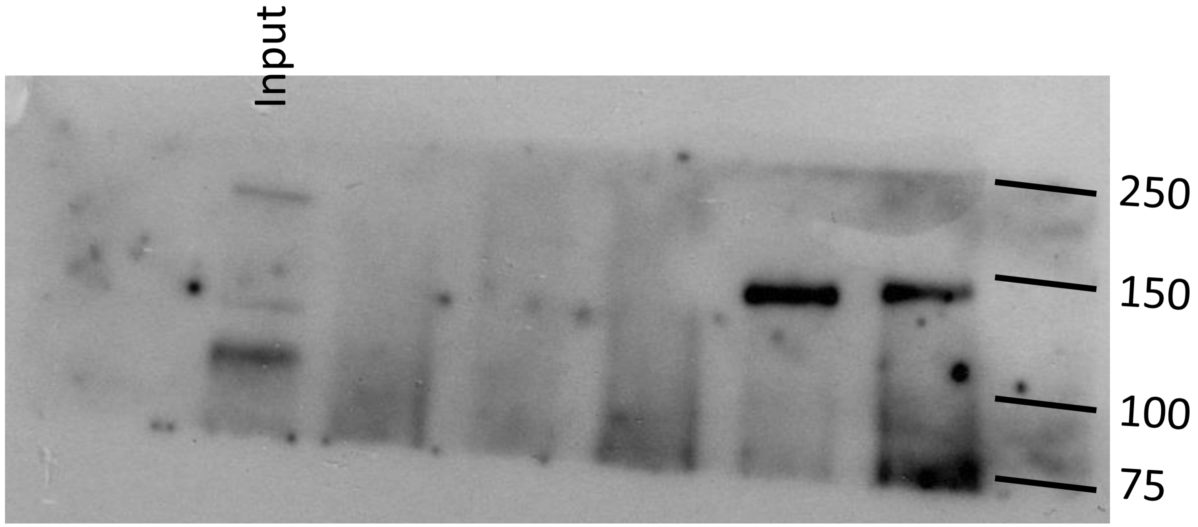

IP: anti-HIF-β	-	+	+	+	+
Rapamycin	-	-	+	-	+
Cetuximab	-	-	-	+	+

Blot probed with anti-HIF-β antibody

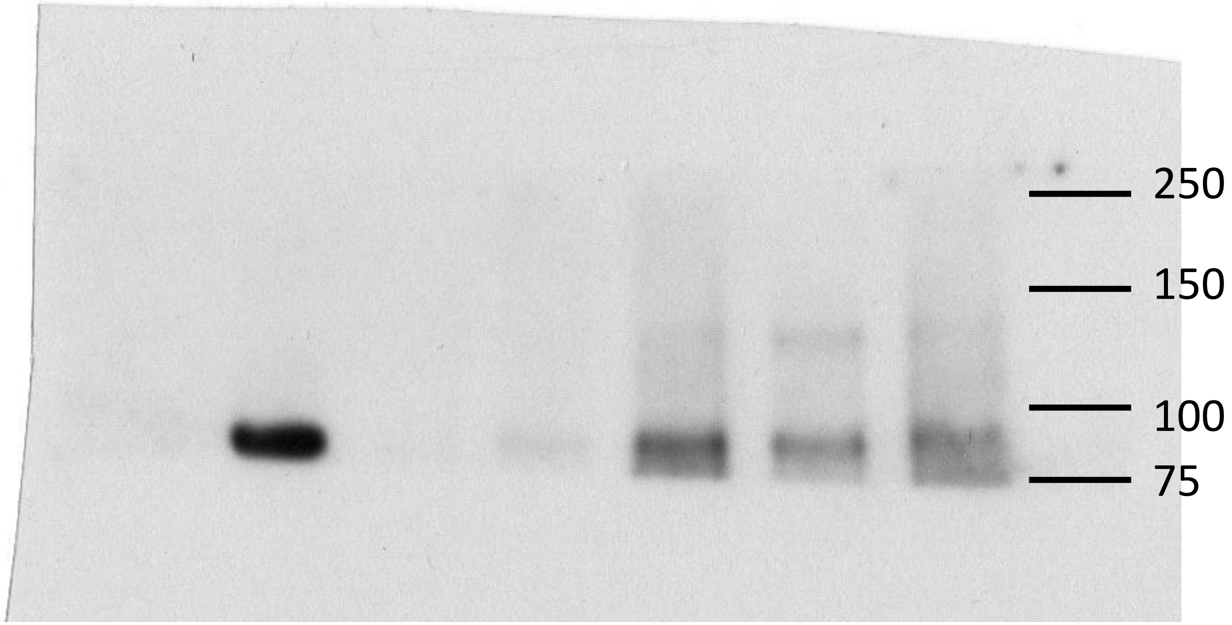

Western blots Figure S1C– Cal27 cell line

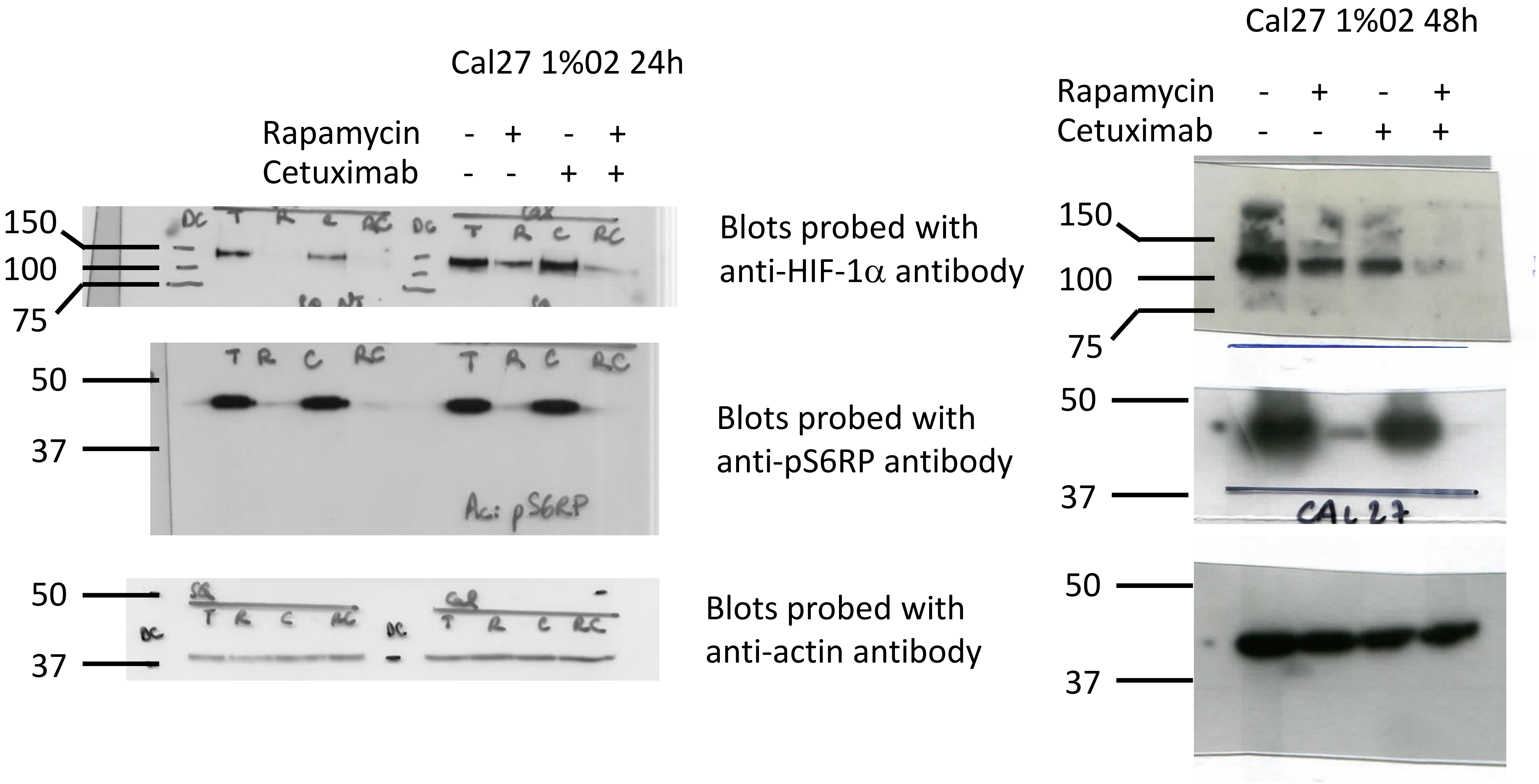

Western blots Figure S1C– SQ20B cell line

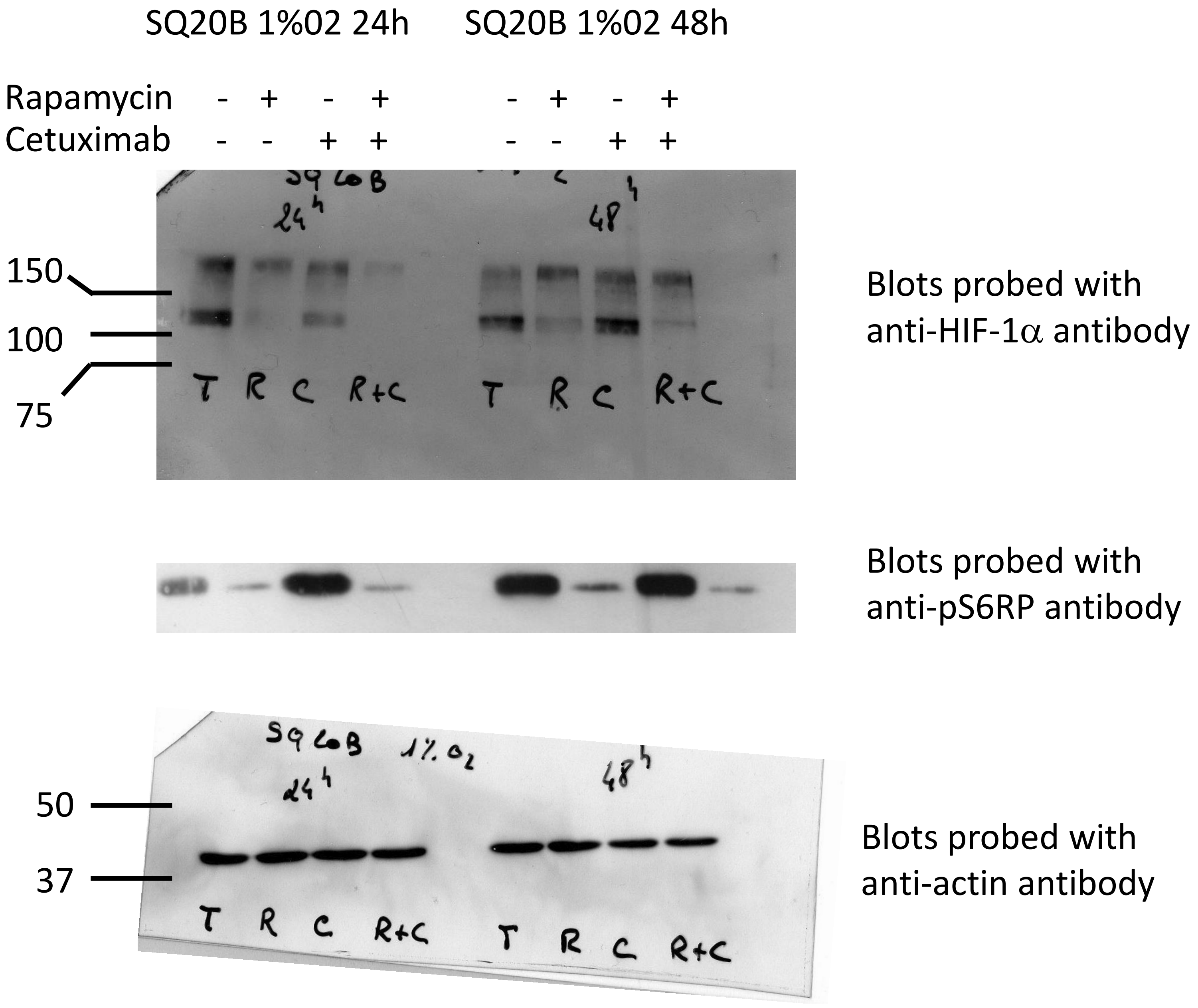

Western blots Figure S1C– Cal27 cell line –S6RP antibody

During the review process, we performed Western blot analyses of whole protein extracts of SQ20B and Cal27 cells by probing membranes with an anti-total S6RP protein. We used the Protein Ladder Prestained (Euromedex) as a molecular weight marker.

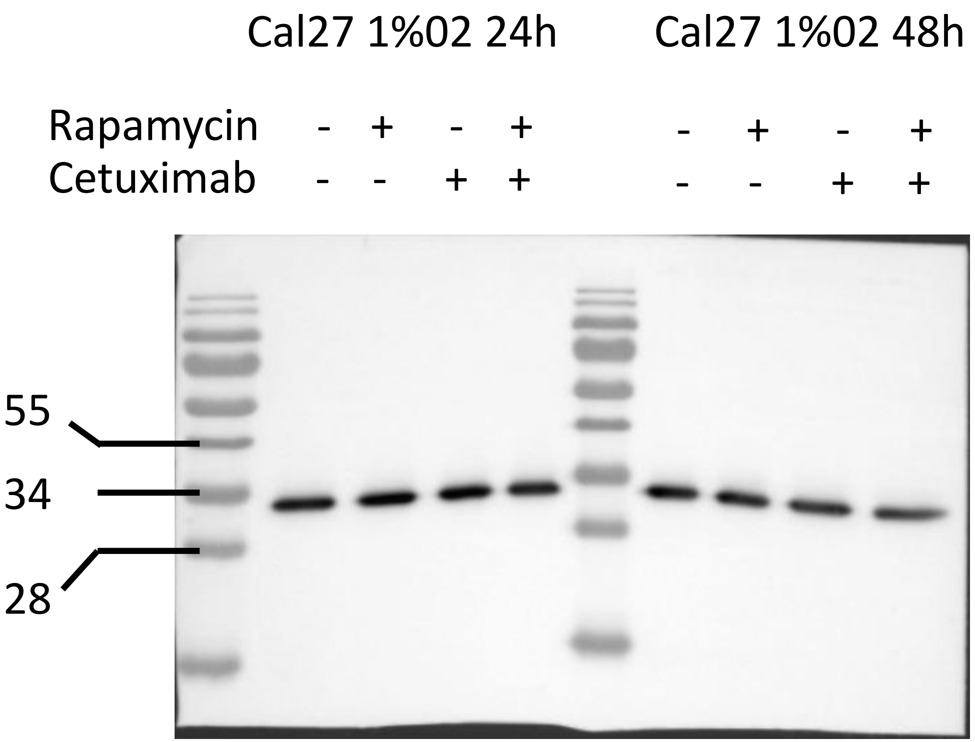

Western blots Figure S1C– SQ20B cell line –S6RP antibody

During the review process, we performed Western blot analyses of whole protein extracts of SQ20B and Cal27 cells by probing membranes with an anti-total S6RP protein. We used the Protein Ladder Prestained (Euromedex) as a molecular weight marker.

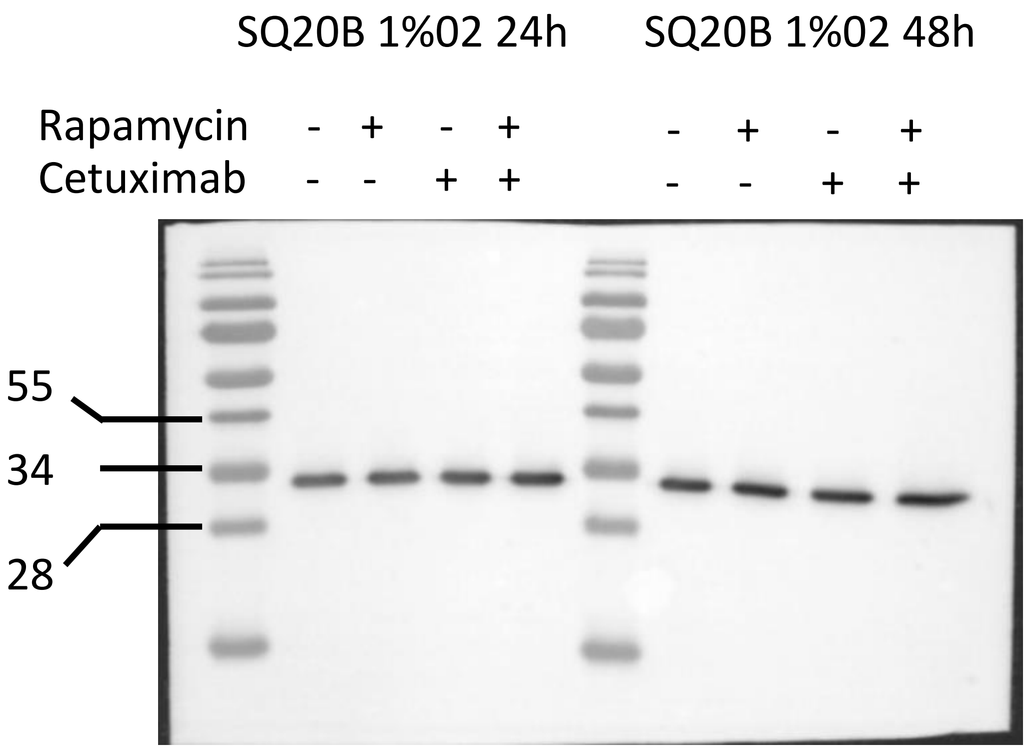

Western blots Figure S5B

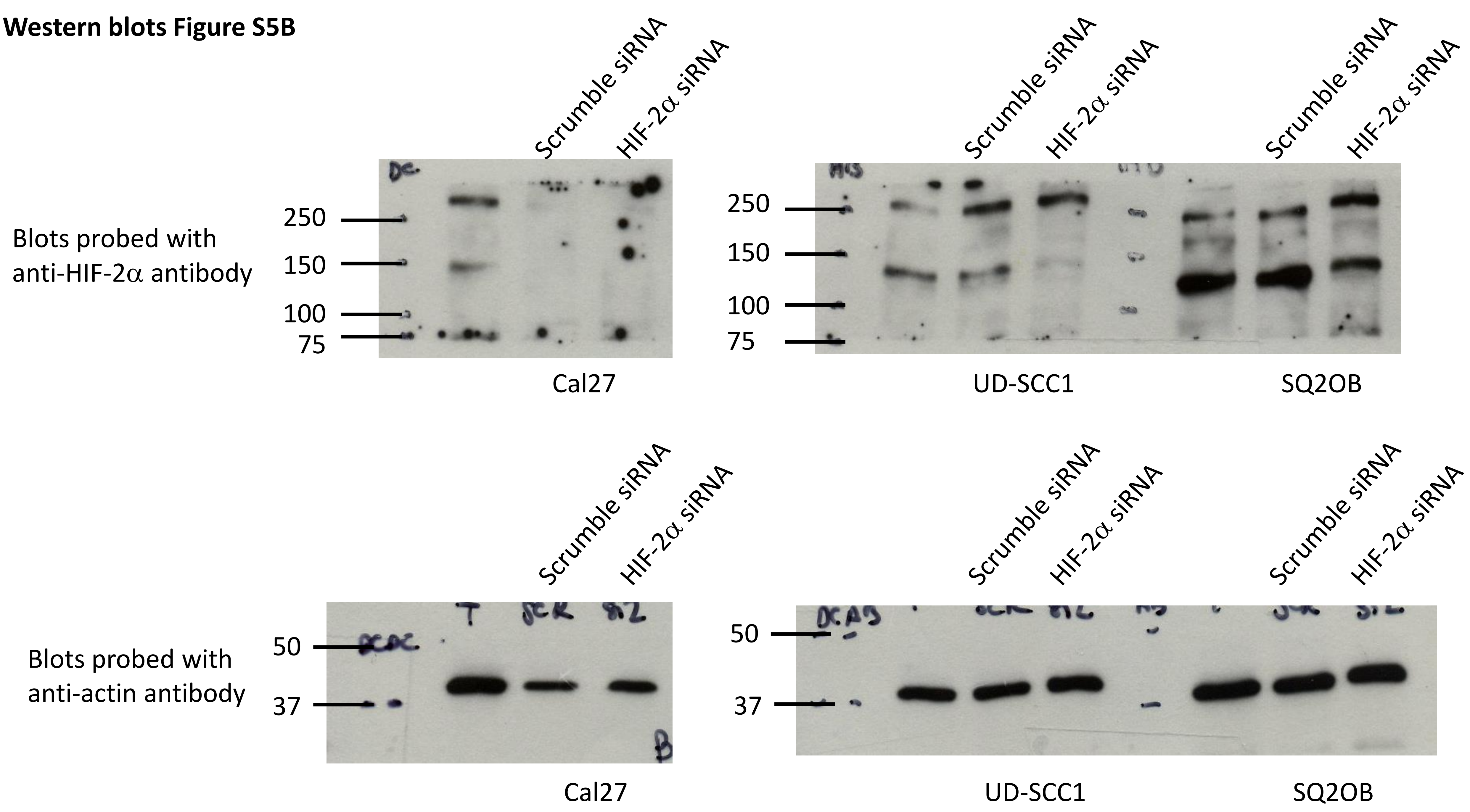

Supplement: Supplementary file 1 [file cancers-11-01607-s001.zip › Western blots-Original pictures.pdf]
